# Supplementary figures and images for: HspB8 prevents aberrant phase transitions of FUS by chaperoning its folded RNA-binding domain
Source: eLife. 2021 Sep 6;10:e69377. doi: 10.7554/eLife.69377 (PMC8510580; doi:10.7554/eLife.69377)

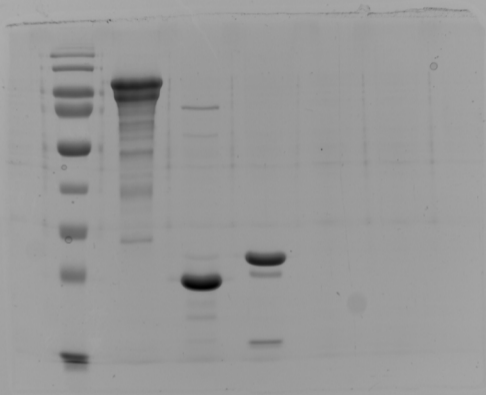

Supplement: Figure 2—figure supplement 1—source data 1. [file elife-69377-fig2-figsupp1-data1.pdf]
